# Microfibrillar-associated protein 2 is a prognostic marker that correlates with the immune microenvironment in glioma

**DOI:** 10.3389/fgene.2022.989521

**Published:** 2022-09-20

**Authors:** Wanzhen Xu, Ren Geng, Yao Zhao, Xiaoshan Ma, Yang Bai, Yining Jiang, Liyan Zhao, Yunqian Li

**Affiliations:** ^1^ Department of Neurosurgery, First Hospital of Jilin University, Changchun, China; ^2^ Department of Clinical Laboratory, Second Hospital of Jilin University, Changchun, China

**Keywords:** microfibrillar-associated protein 2, glioma, prognostic biomarker, immune microenvironment, extracellular matrix

## Abstract

**Aims:** microfibrillar-associated protein 2 (MFAP2), a component of the extracellular matrix, plays key roles in regulating growth factor signal transduction and various malignant tumors. However, the clinicopathological features of microfibrillar-associated protein 2 in gliomas have not been elucidated to date.

**Methods:** TCGA and CGGA databases were used to study the expression of microfibrillar-associated protein 2 in glioma and its relationship with clinicopathological features of patients with glioma. Western blotting was performed to detect the expression of microfibrillar-associated protein 2 protein in tissue samples from glioma patients. Gene set enrichment analysis (GSEA) was applied to detect biological processes and signal pathways related to microfibrillar-associated protein 2. Single-sample gene set enrichment analysis, TIMER 2.0, and TISIDB databases were used to evaluate the role of microfibrillar-associated protein 2 in tumor immune characteristics. The prognostic role of microfibrillar-associated protein 2 in glioma was analyzed using the Kaplan-Meier method and Cox regression. Survival data were used to establish a nomogram prediction model.

**Results:** microfibrillar-associated protein 2 expression was significantly elevated in gliomas. receiver operating characteristic analysis revealed good discrimination of microfibrillar-associated protein 2 between glioma and normal tissues. High expression of microfibrillar-associated protein 2 was associated with malignant phenotypes, such as histological type. Based on gene set enrichment analysis, we identified pathways associated with high microfibrillar-associated protein 2 expression. High microfibrillar-associated protein 2 expression was related to the infiltration of tumor immune cells, including Th2 cells and macrophages, and correlated with key markers of T-cell exhaustion. Based on the TISIDB database, microfibrillar-associated protein 2 was observed to be associated with chemokines, chemokine receptors, and multiple immunoinhibitors in glioma. Kaplan–Meier survival analyses revealed that high microfibrillar-associated protein 2 expression predicted poor overall survival, DSS, and PFS in patients with glioma. By combining microfibrillar-associated protein 2 and other prognostic factors, a nomogram prognostic prediction model was constructed, which demonstrated an ideal prediction effect.

**Conclusion:** microfibrillar-associated protein 2 is a potential prognostic marker that plays a key role in glioma development given its association with malignant phenotypes, cancer-related pathways and tumor immunity.

## Introduction

As the most common intracranial primary tumor, glioma exhibits aggressive behavior and is associated with high disability and mortality rates (Ostrom et al., 2022). The World Health Organization (WHO) classifies gliomas according to their degree of malignancy, molecular markers and pathological features. (Louis et al., 2021). Additionally, newly identified molecular biomarkers have become increasingly important in defining diagnostic information and influencing clinical decision-making of gliomas. Indeed, significant tumor biomarkers are needed to clarify the molecular mechanisms underlying glioma occurrence and development (Kan et al., 2020).

Extracellular matrix (ECM) is a highly dynamic network comprising collagen, proteoglycans, glycosaminoglycans, elastin, fibronectin and several other glycoproteins (Theocharis et al., 2016). In normal and tumor tissues, ECM constituents and cell adhesion receptors bind to one another to constitute intricate cell scaffolds for cell residence. In addition, ECM acts as a storage and binding site of bioactive molecules. Signals from ECM are transmitted to cells via cell surface receptors and regulate various cell functions, such as survival, proliferation, migration, differentiation, and immune response, to maintain normal homeostasis (Brown, 2011; Humphrey et al., 2014). A growing body of research has demonstrated that ECM remodeling plays a major role in shaping the inflammatory and immune milieu of tumors. ECM plays multiple roles in regulation of the tumor immune cycle, including inhibiting cancer cell death, reducing the deliverance of cancer cell antigens, interfering with cancer antigen submission, triggering and activating of effector T cells, regulating T cell migration and regulating perturbations in identification and destruction of cancer cells by T cells (He et al., 2021).

Microfibrillar-associated proteins (MFAPs) are a group of ECM glycoproteins comprising components of ECM microfibrils that are involved in microfibrillar-assembly elastin production and tissue environmental stability (Zhu et al., 2021). MFAPs include five subfamily members (MFAP1-5), among which the MFAP2-encoding gene located at 1p36.13 was the first family member to be characterized. MFAP2 functions to regulate growth factor signal transduction (Craft et al., 2018). MFAP2 was recently reported to be associated with various malignant tumors. For example, as a prognostic marker of hepatocellular carcinoma, MFAP2 leads to hepatocellular carcinoma cell epithelial-mesenchymal transition, and also promotes hepatocellular carcinoma angiogenesis via vascular endothelial growth factor A (Zhang et al., 2021); MFAP2, an oncogene in gastric cancer, promotes the progression of cancer through the integrin α5β1/FAK/ERK1/2 signaling pathway (Yao et al., 2020); MFAP2 is highly expressed in melanoma and leads to melanoma invasion and migration by upregulating EMT-related proteins and Wnt/β-catenin signal pathway (Chen et al., 2020). However, the expression and effection of MFAP2 on gliomas have yet to be reported. Based on the current literature, we hypothesized that MFAP2 plays an important role in malignant phenotype of glioma invasion, metastasis, and immunosuppression by remodeling tumor-related ECM components.

In this study, we investigated the influence of MFAP2 in patient survival and the correlation between MFAP2 expression and clinicopathological elements of glioma. To this end, we harnessed RNA-seq data from the CGGA and TCGA databases and enriched signaling pathways related to MFAP2 through bioinformatics analysis. Furthermore, we investigated the prognostic value of MFAP2 in glioma using a nomogram model. Our analyses revealed a correlation between MFAP2 and immune cell infiltration in gliomas. It affords crucial insight into the roles of MFAP2 in glioma and provides new directions for exploring the occurrence and development mechanism of glioma.

## Materials and methods

### Western blotting

Tissue samples (Neurosurgery department, The First Hospital of Jilin University), including 16 pairs of gliomas and para-cancer tissues, were collected with the approval of the Research Ethics Committee of the First Hospital of Jilin University. They were lysed using RIPA buffer (Beyotime Biotechnology, Shanghai, China) supplemented with protease inhibitors. The concentration of the protein sample was calculated using the BCA protein analysis kit (Thermo Scientific MA, United States) as per the manufacturer’s instructions. The proteins in the sample (containing 20 μg total protein) were separated using SDS-PAGE and transferred onto PVDF membrane (Merck Micropore, Burlington, MA, United States). Next, the membrane was blocked with 5% non-fat dried milk in TBS at 20°C for 90 min. Then, the membrane was incubated overnight with primary antibodies at 4°C. Primary antibodies for MFAP2 and β-actin were purchased from Abmart (Shanghai, China). Thereafter, incubation with appropriate horseradish peroxidase-conjugated secondary antibodies was carried out at 20°C for 2 h. The membrane was visualized using a gel imaging system (Sage Creation Science Co. Ltd., Beijing, China).

### Data collection and preprocessing

RNA-seq data in transcripts per million formats for TCGA (https://www.cancer.gov/tcga.) and GTEx (http://www.gtexportal.org) were harmonized using the Toil process in the UCSC Xena browser (https://xenabrowser.net/datapages/) (Vivian et al., 2017). Data were collected from GBMLGG of TCGA (689 cases of glioma) and corresponding normal tissues in GTEx (1,157 cases). Gene expression data for CGGA were extracted from GlioVis (http://gliovis.bioinfo.cnio.es/) (Bowman et al., 2017; Zhao et al., 2021). We first excluded patients with missing prognostic information or unknown clinical features. Data were divided into high- and low-expression groups according to the median expression of MFAP2. This study was carried out in accordance with the Declaration of Helsinki.

### DEG analysis

Expression profiles (HTSeq-Counts) were compared using the R package DESeq2 (1.26.0) (Love et al., 2014) to identify DEGs between the high- and low-MFAP2 expression groups. A |log2 (FC)| >2 and adjusted *p*-value <0.05 were considered threshold values for DEGs.

### The enrichment analysis on the DEGs

We used the cluster profiler R package (v3.14.3) to perform Gene Ontology (GO) enrichment analysis, Kyoto Encyclopedia of Genes and Genomes (KEGG) pathway enrichment analysis, and GSEA enrichment analysis on the DEGs (Yu et al., 2012). In GSEA analysis, MSigDB category (c2. cp.v7.2. symbols.gmt) was used as the reference gene set (http://software.broadinstitute.org/gsea/msigdb). *p*-value <0.05 and q < 0.25 were considered as statistically significant.

### Immune infiltration analysis

The ssGSEA method of GSVA (Hanzelmann et al., 2013) was applied to investigate the correlation between MFAP2 and the 24 immunocyte types. The signature of characteristic genes of various immune cells was derived from a previous study (Bindea et al., 2013). Spearman’s correlation was applied to analyze correlation between MFAP2 expression and glioma immunocytes. Wilcoxon rank sum test was applied to investigate the degree of glioma immunocytes infiltration in high- and low-MFAP2 expression groups.

### TIMER 2.0 and TISIDB database

TIMER 2.0 (http://timer.comp-genomics.org/) was used to adjust the effects of tumor purity on gene expression and to perform relevant analysis (Li et al., 2017). TISIDB (http://cis.hku.hk/TISIDB/) was applied to analyze the correlation between MFAP2 and chemokine receptors, chemokines, and immunosuppressants (Ru et al., 2019).

### Statistical analysis of clinical factors and prognosis

Statistical analyses were analyzed using R (version 3.6.3). MFAP2 expression levels in tumor and unpaired normal tissues (TCGA tumor vs TCGA normal + GTEx) were compared using the Wilcoxon rank-sum test. Wilcoxon rank-sum test and receiver operating characteristic (ROC) analysis were applied to compare MFAP2 expression in tumor and normal tissues. ROC analysis was performed with pROC (1.17.0.1) to estimate the ability of MFAP2 to differentiate glioma from normal tissues. Normal, corrected Chi-square, Wilcoxon rank-sum, and Kruskal–Wallis rank-sum tests were used to analyze the relationship between MFAP2 expression and clinicopathological features. Analysis of survival was implemented via a Cox proportional hazards model and Kaplan-Meier method. Hazard ratio (HR) of overall survival (OS) was estimated using univariate and multivariate Cox regression analysis. Variables with *p* < 0.05 in univariate Cox regression analysis were included in multivariate Cox regression analysis. Kaplan-Meier survival curves were drawn using Survminer (ver0.4.9, https://cran.r-project.org/web/packages/survminer/index.html).

### Prognostic model construction

The independent predictive factors of glioma were established by univariate and multivariate Cox regression analysis. RMS (6.2–0, https://cran.rproject.org/web/packages/rms/index.html) and SURVIVAL (3.3–1, https://cran.r-project.org/web/packages/survival/index.html) were used to construct nomogram, and the calculation was repeated 200 times using the bootstrap method. Each group of 40 samples was used to verify the nomogram and to draw a correction curve.

## Results

### Clinical characteristics of microfibrillar-associated protein 2 in glioma

**FIGURE 1:**
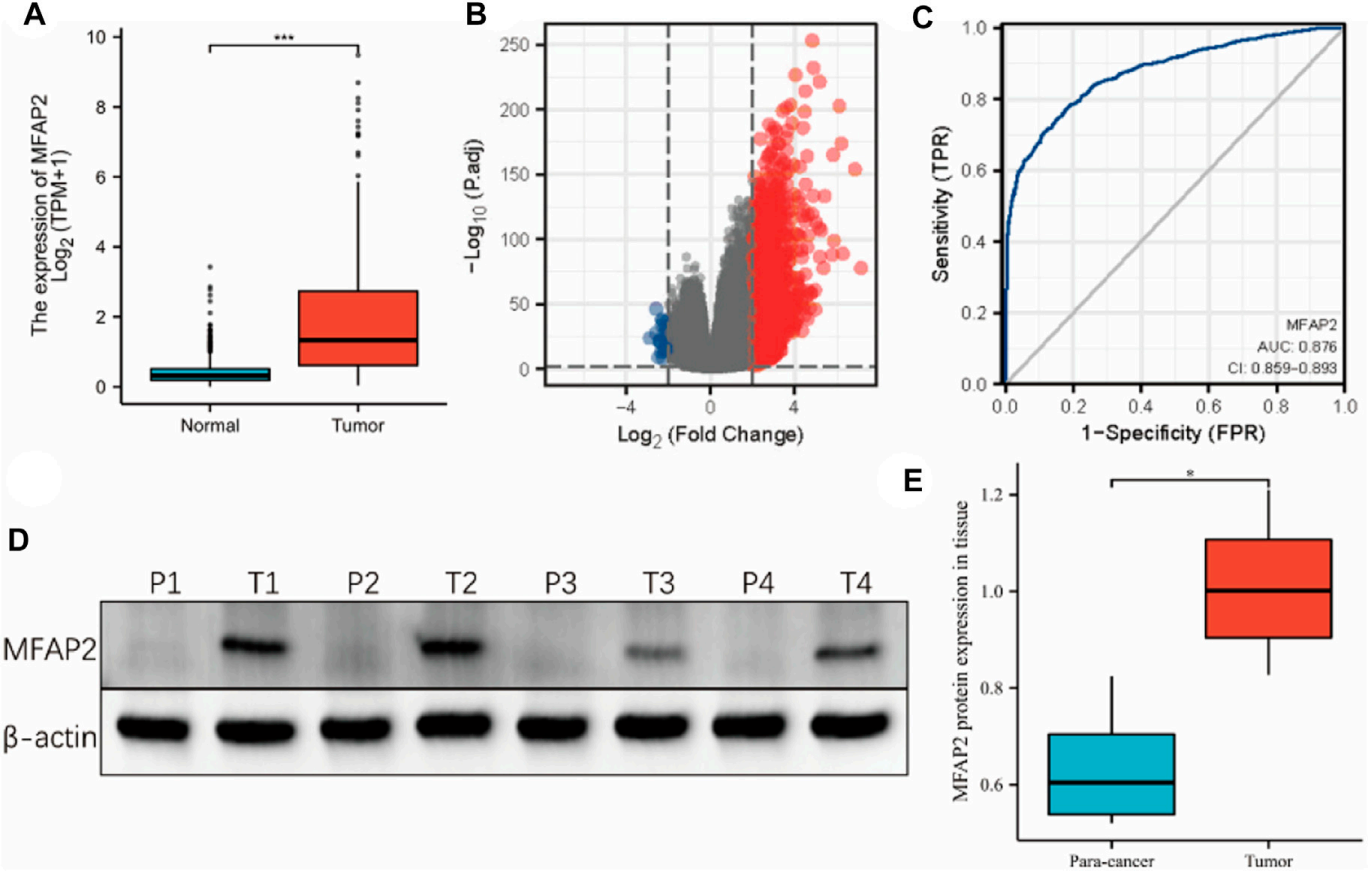
Expression of MFAP2 in tumor and normal tissues of patients with glioma. **(A)** Higher MFAP2 expression in glioma compared to that in normal tissue from the TCGA-GTEx database. **(B)** DEGs between the high- and low-MFAP2 expression groups. **(C)** ROC analysis of MFAP2 revealed promising differentiation ability between tumor and normal tissues. **(D)** and **(E)** Representative Western blot and the corresponding expression levels of MFAP2 protein in para-cancer and tumor tissues. *p*-value significance codes: ***p ≤ 0.001, **p ≤ 0.01, *p ≤ 0.05.

We obtained clinicopathological characteristics of patients with glioma from the TCGA database (Table 1). The relationship between MFAP2 expression and clinical characteristics was analyzed using the Chi-square and Wilcoxon rank-sum tests. High MFAP2 expression levels were significantly associated with WHO grade, IDH status, histological type, 1p/19q co-deletion, primary therapy outcome, and age, *p* < 0.001, but not gender (*p* = 0.491) or race (*p* = 0.246). Contrastive research of 1,157 cases of normal tissues and 689 cases of glioma tissues in the TCGA-GTEx database showed that MFAP2 expression levels were significantly higher in glioma than in normal tissues (*p* < 0.001, Figure 1A). A total of 1741 DEGs between the high- and low-MFAP2 expression groups were identified, including 1715 upregulated genes and 26 downregulated genes (Figure 1B). Furthermore, MFAP2 overexpression exhibited a strong ability to differentiate glioma from normal tissue, with an AUC value of 0.876 (Figure 1C). To study the expression of MFAP2 protein in glioma, expression of MFAP2 protein was assessed in freshly isolated para-cancer and tumor tissues. It was found that the expression of MFAP2 protein in glioma tissues was higher than that in the para-cancer tissues (Figures 1D,H).

**TABLE 1:** The characteristics of patients with glioma based on TCGA.

Characteristic	Low expression of MFAP2	High expression of MFAP2	*p*	Statistic	Method
n	348	348			
WHO grade, n (%)			<0.001	225.32	Chisq.test
G2	176 (27.7%)	48 (7.6%)			
G3	126 (19.8%)	117 (18.4%)			
G4	4 (0.6%)	164 (25.8%)			
IDH status, n (%)			<0.001	201.79	Chisq.test
WT	34 (5%)	212 (30.9%)			
Mut	311 (45.3%)	129 (18.8%)			
1p/19q codeletion, n (%)			<0.001	94.58	Chisq.test
codel	142 (20.6%)	29 (4.2%)			
non-codel	206 (29.9%)	312 (45.3%)			
Primary therapy outcome, n (%)			<0.001	19.78	Chisq.test
PD	54 (11.7%)	58 (12.6%)			
SD	101 (21.9%)	46 (10%)			
PR	47 (10.2%)	17 (3.7%)			
CR	100 (21.6%)	39 (8.4%)			
Gender, n (%)			0.491	0.48	Chisq.test
Female	154 (22.1%)	144 (20.7%)			
Male	194 (27.9%)	204 (29.3%)			
Race, n (%)			0.246	2.81	Chisq.test
Asian	4 (0.6%)	9 (1.3%)			
Black or African American	14 (2%)	19 (2.8%)			
White	323 (47.3%)	314 (46%)			
Age, n (%)			<0.001	31.68	Chisq.test
≤60	307 (44.1%)	246 (35.3%)			
>60	41 (5.9%)	102 (14.7%)			
Histological type, n (%)			<0.001	216.65	Chisq.test
Astrocytoma	107 (15.4%)	88 (12.6%)			
Glioblastoma	4 (0.6%)	164 (23.6%)			
Oligoastrocytoma	88 (12.6%)	46 (6.6%)			
Oligodendroglioma	149 (21.4%)	50 (7.2%)			
Age, meidan (IQR)	41 (32, 52)	52 (37, 62.25)	<0.001	41,863	Wilcoxon

Note. WT, Wild type. Mut, mutant. PD, progressive disease; SD, stable disease; PR, partial response; CR, complete response.

### Upregulation of microfibrillar-associated protein 2 in malignant phenotypes of glioma

**FIGURE 2:**
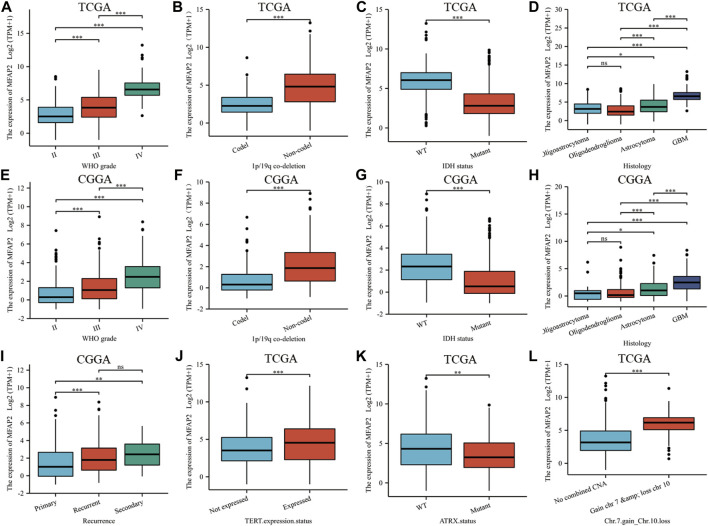
The association between MFAP2 expression and clinicopathologic features. MFAP2 expression patterns for different grades of glioma in TCGA **(A)** and CGGA **(E)** database. MFAP2 expression patterns for different co-deletions of 1p/19q in glioma in TCGA **(B)** and CGGA **(F)** database. MFAP2 expression patterns for different IDH status of glioma in TCGA **(C)** and CGGA **(G)** database. MFAP2 expression patterns for different histological types of glioma in TCGA **(D)** and CGGA **(H)** database. **(I)** MFAP2 expression patterns for glioma recurrence in CGGA database. **(J–L)** Relationship between MFAP2 expression and TERT expression status **(J)**, ATRT status **(K)**, and chromosomes 7 and 10 **(L)** in TCGA database. Codel, codeletion. Non-codel, non-codeletion. WT, wild type. *p*-value significance codes: ***p ≤ 0.001, **p ≤ 0.01, *p ≤ 0.05. ns, not significant.

According to WHO standards, gliomas are classified as grades I through IV according to their malignant degree, with the most malignant glioma was grade IV. We compared MFAP2 expression levels of gliomas of different grades in TCGA database and observed that MFAP2 positively correlated with glioma grade (Figure 2A). Given the influence of 1p19q co-deletion and IDH status on glioma, we researched the association between these factors and MFAP2 expression. Patients with high MFAP2 expression had a higher proportion of 1p19q non-codeletion (Figure 2B) and wild-type IDH (Figure 2C), which were both signs of poor prognosis. Subsequently, we observed that MFAP2 overexpression was related to the histological type of gliomas and was significantly overexpressed in glioblastoma multiforme (Figure 2D). Similar results were validated using the CGGA database (Figures 2E–H). Based on the CGGA database, we identified that MFAP2 expression was significantly higher in the recurrent and secondary groups than in the primary group (Figure 2I). MFAP2 expression was significantly higher in the TERT expression (Figure 2J) and chromosome seven gains/chromosome 10 loss groups (Figure 2L) but lower in the ATRX mutant group (Figure 2K). These results suggest that MFAP2 is overexpressed in gliomas with malignant phenotypes, which may be associated with the poor prognosis of gliomas.

### The functions of microfibrillar-associated protein 2 in glioma

**FIGURE 3:**
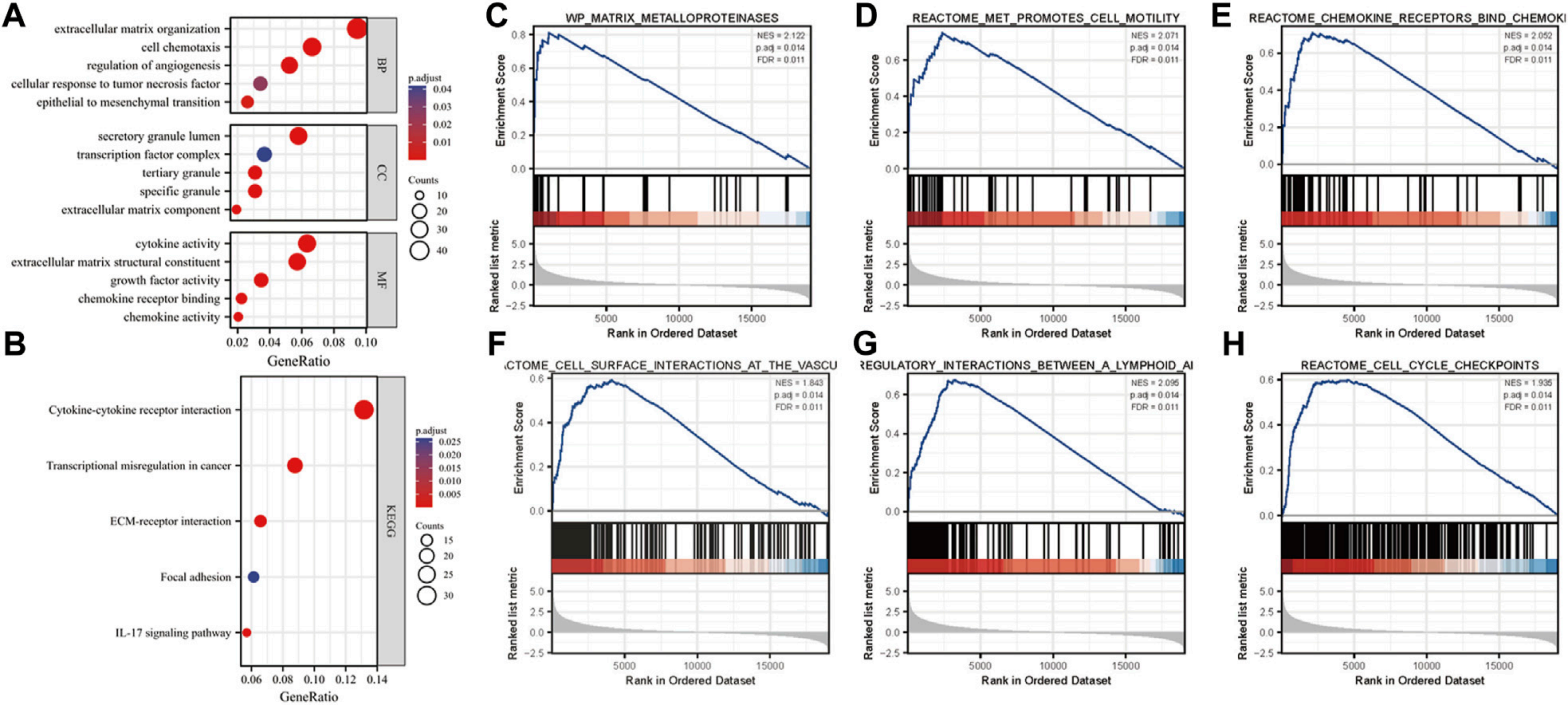
Analysis of the role of MFAP2 in glioma. **(A)** Analysis of the GO results. **(B)** Analysis of the KEGG signaling pathways involving the MFAP2 protein in glioma. **(C–H)** Enrichment plots from GSEA.

**FIGURE 4:**
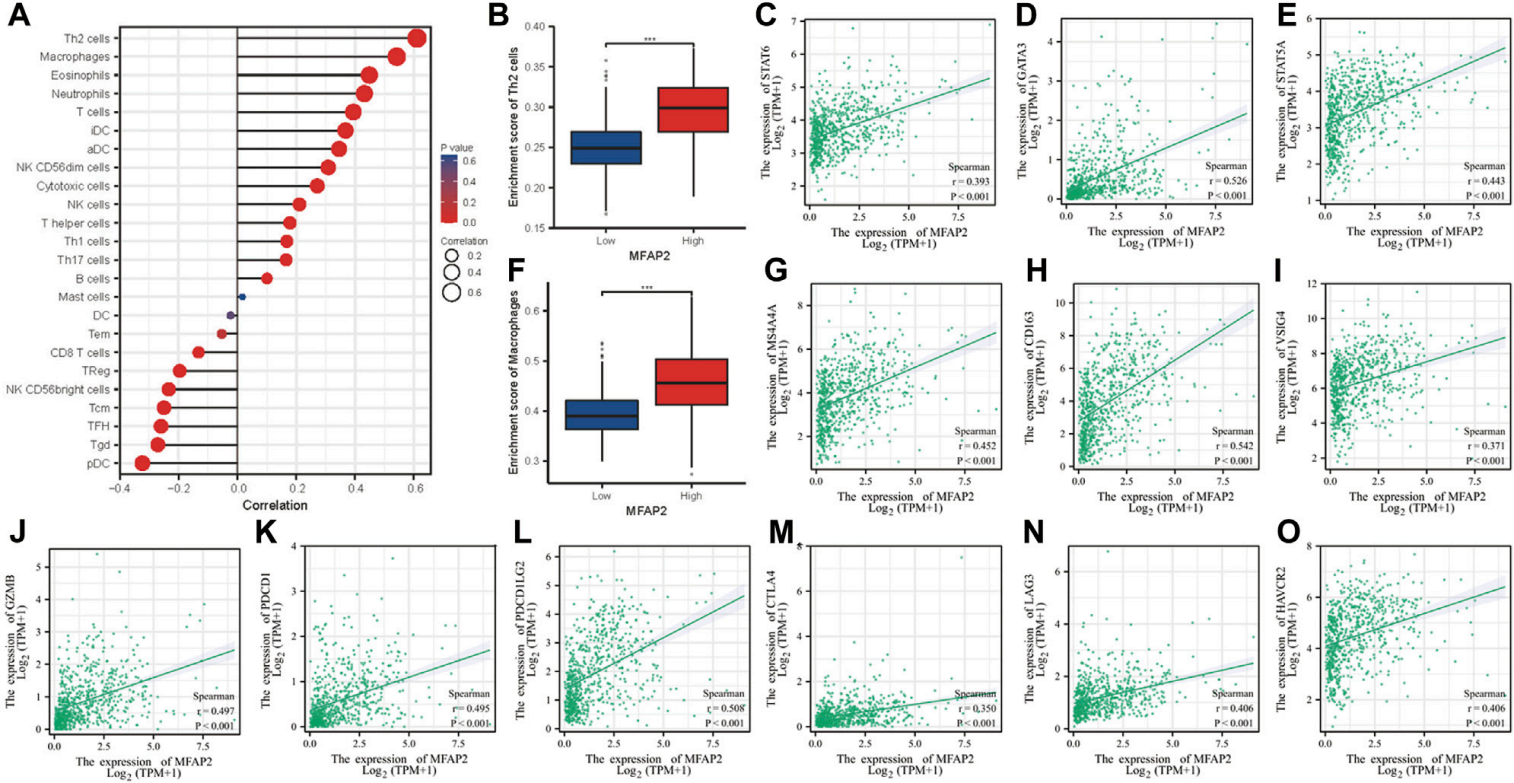
Association between tumor-infiltrating lymphocytes and MFAP2 in glioma. **(A)** Association between abundance of the 24 types of immunocytes and MFAP2 expression level. **(B)** Th2 infiltration level in the high- and low-MFAP2 expression groups in the TCGA cohort. **(C–E)** Correlation between MFAP2 expression and Th2 markers. **(F)** Macrophage infiltration level in the high- and low-MFAP2 expression groups in the TCGA cohort. **(G–I)** Correlation between MFAP2 expression and M2-like macrophage markers. **(J–O)** Correlation between MFAP2 expression and T cell exhaustion markers. *p*-value significance codes: ***p ≤ 0.001.

To explore the role of MFAP2 in gliomas, the GO and KEGG enrichment analyses based on MFAP2 expression were performed. The results confirmed that the biological processes associated with MFAP2 were extracellular matrix organization, cell chemotaxis, regulation of angiogenesis, cellular response to tumor necrosis factor, and epithelial-to-mesenchymal transition. The cellular components where MFAP2 was localized were mostly the secretory granule lumen, transcription factor complex, tertiary granule, specific granule, and the extracellular matrix. The molecular functions of MFAP2 were associated with cytokine activity, extracellular matrix structural support, growth factor activity, chemokine receptor binding, and chemokine activity (Figure 3A). KEGG enrichment results confirmed that MFAP2 was involved in cytokine-cytokine receptor interaction, transcriptional misregulation in cancer, ECM-receptor interaction, focal adhesion, and the IL-17 signaling pathway (Figure 3B). GSEA was used for enrichment analysis of overexpressed MFAP2 to identify activated signaling pathways in gliomas. As presented in Figure 4, six pathways were identified, including the activation of matrix metalloproteinases (Figure 3C), MET promotion of cell motility (Figure 3D), chemokine receptor binding to chemokines (Figure 3E), cell surface interactions at the vascular wall (Figure 3F), immunoregulatory interactions between lymphoid and non-lymphoid cells (Figure 3G), and cell cycle checkpoints (Figure 3H). These results implied that MFAP2 plays an important role in glioma cell migration, proliferation, tumor invasion, angiogenesis and immune infiltration.

### Relationship between microfibrillar-associated protein 2 expression level and immune infiltration

Spearman’s correlation coefficient was used to investigate the relationship between MFAP2 expression level and immune cell enrichment. MFAP2 expression level was positively correlated with Th2 cells, macrophages, eosinophils, neutrophils and T cells but negatively correlated with pDC, Tgd, and TFH (Figure 4A). In view of the immunosuppressive effects of Th2 cells and M2 macrophages in cancer, we investigated the relationship between their infiltration levels and MFAP2 expression. As the result shown, MFAP2 overexpression was correlated with Th2 cells infiltration (Figure 4B). MFAP2 expression levels were positively correlated with STAT6 (*p* < 0.001, r = 0.393) (Figure 4C), GATA3 (*p* < 0.001, r = 0.526) (Figure 4D), and STAT5A (*p* < 0.001, r = 0.443) (Figure 4E) in Th2 cells. In addition, MFAP2 overexpression was correlated with macrophages (Figure 4F). And at the same time, MFAP2 expression levels were positively correlated with key markers of M2 macrophages, including MS4A4A (*p* < 0.001, r = 0.452) (Figure 4G), CD163 (*p* < 0.001, r = 0.542) (Figure 4H), and VSIG4 (*p* < 0.001, r = 0.371) (Figure 4I). Moreover, MFAP2 expression levels were positively correlated with key markers of T-cell exhaustion, including GZMB (*p* < 0.001, r = 0.497) (Figure 4J), PDCD1 (*p* < 0.001, r = 0.495) (Figure 4K), PDCD1LG2 (*p* < 0.001, r = 0.508) (Figure 4L), CTLA4 (*p* < 0.001, r = 0.350) (Figure 4M), LAG3 (*p* < 0.001, r = 0.406) (Figure 4N), and HAVCR2 (*p* < 0.001, r = 0.406) (Figure 4O). Given the effects of tumor purity on the microenvironment in glioma, TIMER algorithm was used to correct the expression levels of key immune cell markers affecting tumor purity, and its correlation with MFAP2 was analyzed. We observed that MFAP2 was strongly linked to the majority of key markers of immune-infiltrating cells in LGG. MFAP2 expression in GBM did not exhibit a clear association with infiltrating immune cells (Supplementary Table S1).

**FIGURE 5:**
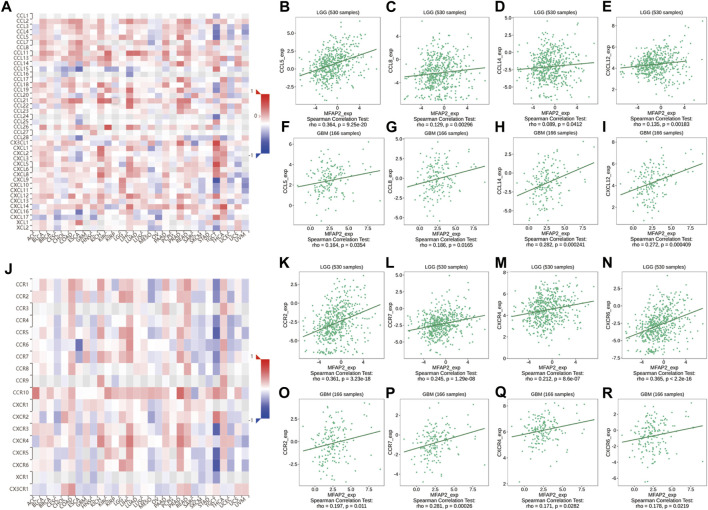
Correlation between MFAP2 expression and chemokines or chemokine receptors in glioma from the TISIDB database. **(A)** Relevance between MFAP2 expression and chemokines in 30 tumors, including LGG and GBM. **(B–E)** Relevance between MFAP2 expression and chemokines (CCL5, CCL8, CCL14, and CXCL13) in LGG. **(F–I)** Correlation between MFAP2 expression and chemokines (CCL5, CCL8, CCL14, and CXCL13) in GBM. **(J)** Correlation between MFAP2 expression and chemokine receptors in 30 tumors, including LGG and GBM. **(K–N)** Correlation between MFAP2 expression and chemokine receptors (CCR3, CCR7, CXCR4, and CXCR6) in LGG. **(O–R)** Correlation between MFAP2 expression and chemokine receptors (CCR3, CCR7, CXCR4, and CXCR6) in GBM. Color images are available online.

Chemokines are essential modulators of the inflammatory response and play a major role in controlling the degree of immune cell infiltration. We identified an association between MFAP2 expression and chemokines; indeed, chemokines such as CCL5, CCL8, CCL14, and CXCL13 were associated with MFAP2 expression in both LGG and GBM. MFAP2 expression was also correlated with chemokine receptors in gliomas, such as CCR3, CCR7, CXCR4, and CXCR6, which were significantly associated with MFAP2 expression in both LGG and GBM (Figure 5).

**FIGURE 6:**
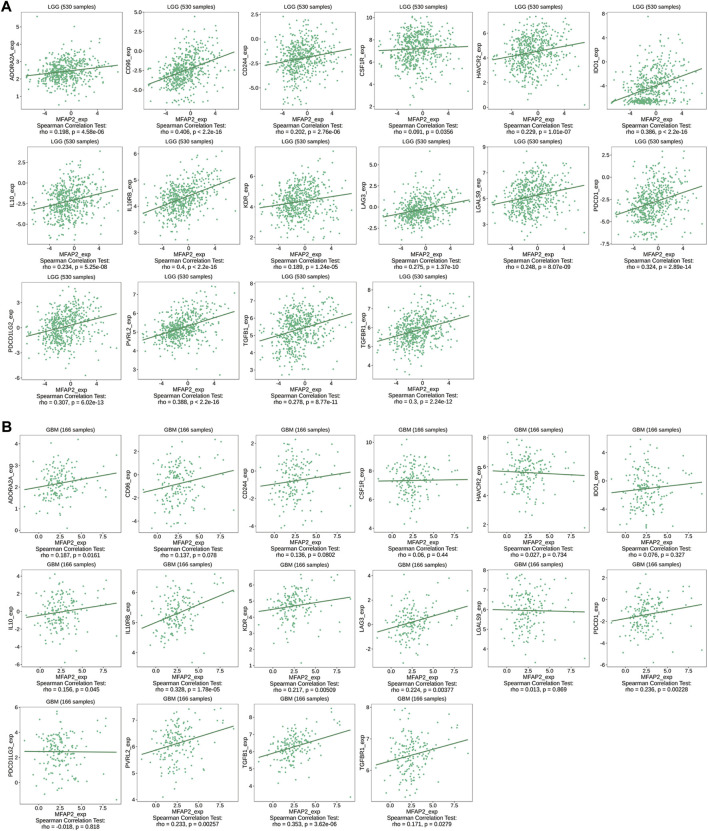
Relevance between MFAP2 expression and immunoinhibitors in glioma in the TISIDB database. Correlation between MFAP2 expression and immunoinhibitors (ADORA2A, CD96, CD244, CSF1R, HAVCR2, IDO1, IL10, IL10RB, KDR, LAG3, LGALS9, PDCD1, PDCD1LG2, PVRL2, TGFB1, and TGFBR1) in LGG **(A)** and in GBM **(B)**. Color images are available online.

Additionally, MFAP2 expression was correlated with immunoinhibitory in gliomas, such as ADORA2A, CD96, CD244, CSF1R, HAVCR2, IDO1, IL10, IL10RB, KDR, LAG3, LGALS9, PDCD1, PDCD1LG2, PVRL2, TGFB1, and TGFBR1, which were positively correlated with MFAP2 expression in both LGG and GBM (Figure 6).

### Prognostic value of microfibrillar-associated protein 2 in glioma

**FIGURE 7:**
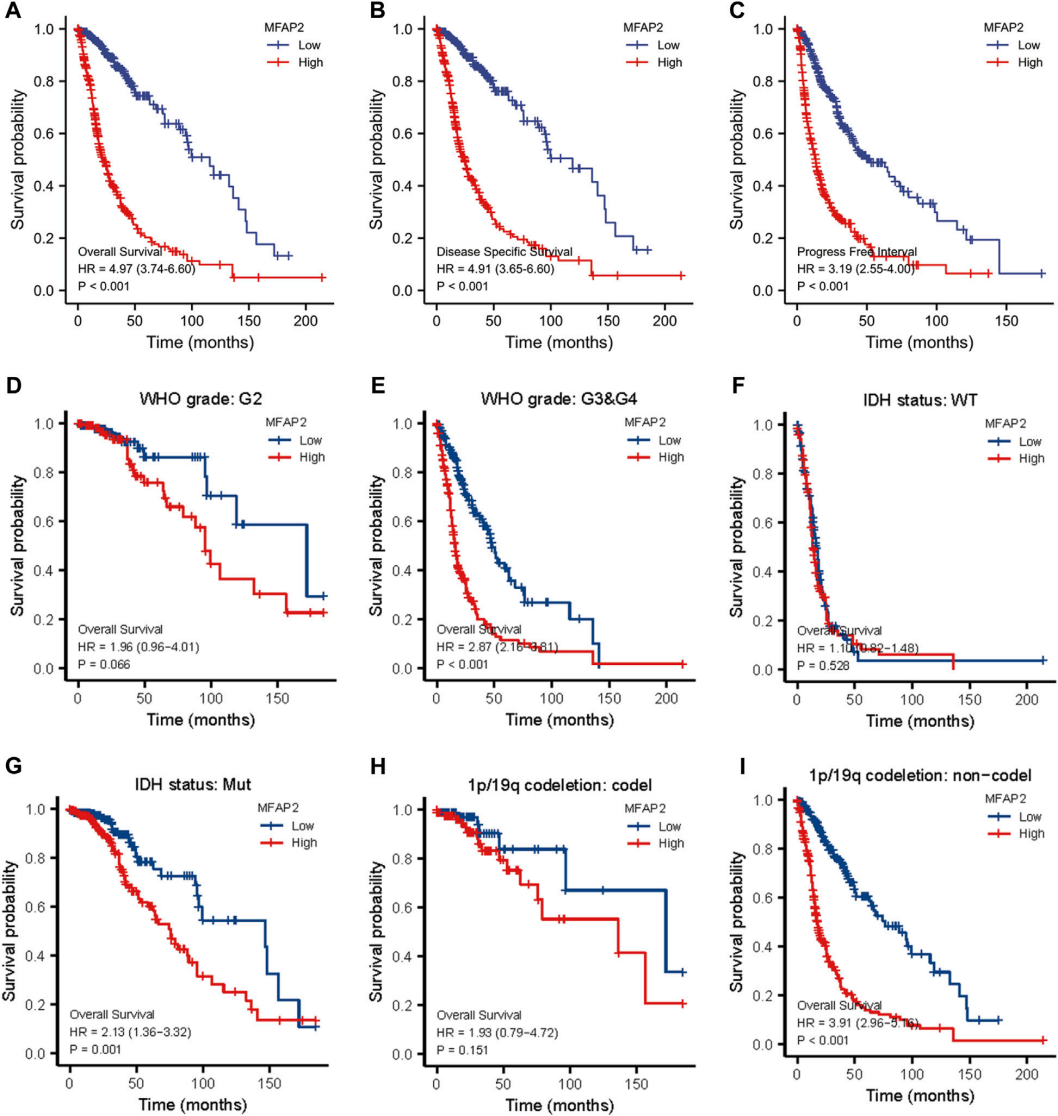
Kaplan–Meier survival analysis of patients with glioma based on MFAP2 expression in the TCGA database. **(A)** Overall survival. **(B)** Disease-specific survival. **(C)** Progression-free survival. **(D)** Overall survival of patients with WHO G2 glioma. **(E)** Overall survival of patients with WHO G3/G4 glioma. **(F)** Overall survival of patients with IDH wild-type glioma. **(G)** Overall survival of patients with IDH mutated glioma. **(H)** Overall survival of patients with 1p/19q codeletion glioma. **(I)** Overall survival of patients with non-codeletion glioma.

To assess the prognostic value of MFAP2 in glioma, Kaplan–Meier survival analysis was performed. As presented in Figure 7A, high MFAP2 expression levels were markedly related to poorer overall survival of patients with glioma. We further verified disease special survival and progression-free interval and observed that both disease special survival and progression-free interval were lower in patients with glioma with high MFAP2 expression than in those with low MFAP2 expression (Figures 7B,C). Multivariate analysis revealed that high MFAP2 expression was associated with low survival in patients with WHO G3/G4 glioma (Figures 7D,E), IDH-mutated glioma (Figures 7F,G) and non-codeletion glioma (Figures 7H,I). These results suggest that patients overexpressing MFAP2 have poorer prognosis, underscoring the potential of MFAP2 as a prognostic biomarker (Table 2).

**TABLE 2:** Cox regression analysis for clinical outcomes in glioma patients.

Characteristics	Total(N)	Univariate analysis		Multivariate analysis	
		Hazard ratio (95% CI)	*p* Value	Hazard ratio (95% CI)	*p* Value
WHO grade	634	References			
G2	223				
G3	243	2.999 (2.007–4.480)	<0.001	2.198 (1.412–3.421)	<0.001
G4	168	18.615 (12.460–27.812)	<0.001	9.242 (2.948–28.967)	<0.001
Primary therapy outcome	461	References			
PD	112				
SD	147	0.440 (0.294–0.658)	<0.001	0.368 (0.230–0.590)	<0.001
PR	64	0.170 (0.074–0.391)	<0.001	0.206 (0.074–0.568)	0.002
CR	138	0.133 (0.064–0.278)	<0.001	0.147 (0.070–0.308)	<0.001
MFAP2	695	References			
Low	347				
High	348	4.971 (3.743–6.602)	<0.001	1.812 (1.202–2.733)	0.005
Age	695	References			
≤60	552				
>60	143	4.668 (3.598–6.056)	<0.001	5.466 (3.326–8.983)	<0.001

Note. WT, Wild type. Mut, mutant. CR, complete response; PD, progressive disease; SD, stable disease; PR, partial response.

### Foundation and verification of a prognostic nomogram that correlated with microfibrillar-associated protein 2 expression

**FIGURE 8:**
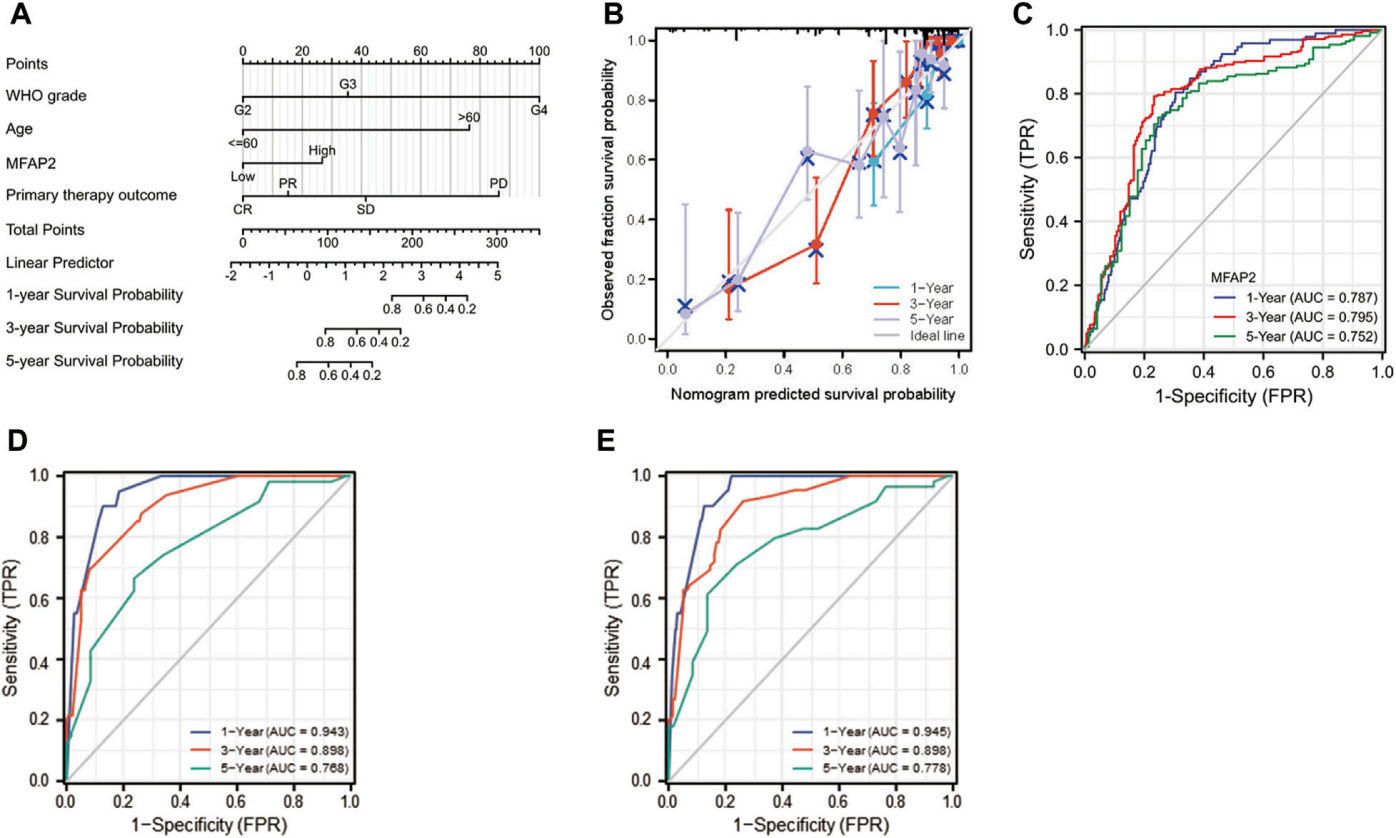
Construction of MFAP2-related prognostic nomogram. **(A)** Nomogram survival prediction in patients with glioma. The top row presents the point value for each variable. **(B)** The calibration curve displays the difference between the model prediction of 1-, 3-, and 5-years survival and actual survival outcomes. **(C)** Time-dependent ROC curve AUC validated the effect of mfAP2-based risk score on prognosis. **(D)** Time-dependent ROC curve AUC validated the effects of WHO grade, primary therapy outcome, age, IDH status, and 1p/19q codeletion based risk score on prognosis. **(E)** Time-dependent ROC curve AUC validated the effect of WHO grade, primary therapy outcome, age, IDH status, and 1p/19q codeletion of the MFAP2-based risk score on prognosis.

To afford a quantitative method to predict the prognosis of glioma patients, we established a nomogram [C-index: 0.859 (0.841–0.877)] by integrating MFAP2 with classical clinical risk factors (Figure 8A). The projected OS rates of patients with glioma at 1, 3, and 5 years were calculated, and the calibration diagram shows that the deviation correction line is close to the ideal curve and nomogram prediction results are in good agreement with actual results (Figure 8B). Time-dependent ROC analyses were performed to identify the prognostic value of MFAP2-based risk scores. AUCs for the 1-, 3-, and 5-years OS predictions for the risk scores were 0.787, 0.795, and 0.752, respectively (Figure 8C). Since WHO grade, primary therapy outcome, age, IDH status, and 1p/19q codeletion are important clinicopathologic characteristics for glioma progression, we further performed ROC analysis combining MFAP2 expression with these parameters. The AUC results for 1-, 3-, and 5-years survival rates were 0.943, 0.898 and 0.768, respectively, for the following parameters: WHO grade, primary therapy outcome, age, IDH status, and 1p/19q codeletion (Figure 8D). On the other hand, the AUC results for 1-, 3-, and 5-years survival rates were 0.945, 0.898 and 0.778, respectively, based on the following parameters: WHO grade, primary therapy outcome, age, IDH status, 1p/19q codeletion and MFAP2 (Figure 8E). Collectively, these results indicated that our nomogram could accurately forecast survival of glioma patients.

## Discussion

Despite recent progress in multimodal treatments for glioma (particularly for the most invasive glioblastoma multiforme), the overall prognosis continue to be unfavorable and the long-term survival rate is very low (Tan et al., 2020). Indeed, the 5-year survival rate for glioma patients is only 6.8% (Ostrom et al., 2021). A principal malignant characteristic of gliomas is their ability to infiltrate. The tumor cells have infiltrated deep when a glioma develops. Studies on the role of tumor microenvironment in glioma invasion have been expanding in recent years. Tumor cells degrade surrounding ECM by secreting protease, resulting in tumor invasion (Tamai et al., 2022). MFAP2 is a key ECM glycoprotein and protein component of ECM microfibrils. Changes in MFAP2 expression regulate ECM remodeling (Gomez de Segura et al., 2021). By interacting with EGFL7, MFAP2 promotes its deposition into fibers in the endothelial ECM, thus playing a key role in vascular development (Villain et al., 2018). Growing evidence suggests that MFAP2 plays a crucial role in the development of malignant tumors. For instance, MFAP2 promotes motility of cancer cells through the integrin α5β1/FAK/ERK pathway in gastric cancer (Yao et al., 2020). In the current study, by analyzing clinicopathological data from patients with glioma, we confirmed for the first time that MFAP2 is associated with the malignant phenotype of glioma, tumor-related pathways, and tumor immunity. Our findings underscore the key role of MFAP2 in glioma development and highlight its potential as a prognostic biomarker.

In the present study, we systematically explored the transcriptome data related to glioma in TCGA and CGGA databases and observed that MFAP2 expression was meaningfully increased in glioma. MFAP2 overexpression exhibited promising discriminative power for distinguishing tumors from normal tissues. Next, we explored MFAP2 expression in various types of glioma and observed that MFAP2 expression levels correlated positively with tumor grade, histology, recurrence, and other biomarkers such as 1p19q codeletion, IDH status, TERT expression, ATRX status, and chromosomes 7 and 10. These outcomes correspond with a former report that MFAP2 promotes epithelial-mesenchymal transformation, proliferation, and migration in tumor cells (Chen et al., 2020; Yao et al., 2020; Zhang et al., 2021). The results of the functional analysis of MFAP2 protein in glioma revealed that MFAP2 correlated with signaling pathways activated in gliomas, such as activation of matrix metalloproteinases, MET promotion of cell motility, chemokine receptor binding to chemokines and cell cycle checkpoints. Mechanistically, MFAP2 overexpression may lead to ECM remodeling, epithelial-mesenchymal transformation, and promotion of tumor cell migration via the integrin α5β1/FAK/ERK (Yao et al., 2020); nevertheless, the specific mechanisms remain unclear.

Furthermore, this study demonstrated that MFAP2 was strongly associated with the degree of immune infiltration in glioma, especially Th2 cells and macrophages. Previous studies have reported that among different tumor-infiltrating lymphocyte subpopulations, two distinct subpopulations of CD4^+^ cells exist, namely, helper T cells (Th)1 and Th2 cells. Th2 cells generate IL-4 and IL-10, which promote tumor growth by inhibiting the host immune system (Dushyanthen et al., 2015; Zhao et al., 2019). Guided by different tumor microenvironment signals, macrophages differentiate into two functional phenotypes: classically activated macrophages (M1) and alternately activated macrophages (M2). Contrary to the antineoplastic effect of M1, M2 has antiphlogistic and oncogenous effects (Yang et al., 2020). By further assaying the correlation between MFAP2 expression levels and immune cells, we observed that the improved MFAP2 expression correlated positively with markers of Th2 cells and M2 macrophages. In addition, an increase in MFAP2 expression correlated positively with markers of T cell exhaustion. The association between MFAP2 and immune cells suggests that MFAP2 plays a pivotal role in the regulation of tumor immunity in gliomas. The relationship between high MFAP2 expression and immune cell infiltration in glioma was further confirmed by an analysis of key immune cell markers. However, TIMER2.0 database analysis revealed that MFAP2 in LGG was significantly associated with immune-infiltrating cells, but not with GBM. This may be because tumor cells adopt different strategies to inhibit the immune system at different stages of the antitumor immune response to survive (Liu and Cao, 2016). We further researched the relationship between MFAP2 expression and chemokines, chemokine receptors, and immunoinhibitors in gliomas. We observed that these parameters were correlated in both LGG and GBM. These results suggest that high MFAP2 expression in gliomas plays a pivotal role in immunosuppression and that targeting MFAP2 is a potential therapeutic strategy to improve the outcome of immunotherapy.

To predict the prognosis of glioma, predictive risk models have increasingly been established, and these different roles in the prognostic risk assessment of glioma patients (Qu et al., 2020; Qu et al., 2021a). Among the clinicopathological parameters, age, histopathology, IDH status, 1p/19q status, radiotherapy, chemotherapy, and recurrence were identified as independent prognostic factors for patients diagnosed with high-grade glioma. The visual prediction model on which these findings are based has been reported to exhibit strong predictive value (Qu et al., 2021b). Recent progress in sequencing technology has promoted in-depth investigations into the molecular diagnosis of gliomas, revealing several genetic biomarkers (Hu and Qu, 2021; Mao et al., 2022). The diagnostic and prognostic value of biomarkers in glioma was confirmed, and some biomarkers were used to guide the diagnosis and treatment of glioma in the fifth edition of the classification of central nervous system tumors released by WHO in 2021 (Louis et al., 2021). Before our study, there have been a lot of prognostic markers in glioma. However, glioma is a kind of malignant tumor with multigene abnormalities. Its pathogenesis may be the high expression of proto-oncogenes and/or the inactivation as well as deletion of tumor suppressor genes, leading to the proliferation and malignant transformation of malignant cells (Seidu et al., 2017). Consequently, when it comes to predicting patients’ prognoses, one biomarker is not sufficient and multiple biomarkers are required (Qu et al., 2021a). In this study, Kaplan-Meier survival and Cox regression analysis showed that high MFAP2 expression levels predicted poor OS, DSS, and PFS in patients with glioma. Common clinicopathological parameters combined with the analysis of MFAP2 expression improve the predictive ability of OS in glioma patients. Moreover, the nomogram based on multivariate Cox analysis could predict short- or long-term survival of patients with glioma. Collectively, these findings highlight the potential of MFAP2 as a novel prognostic biomarker and a potential target for glioma immunotherapy.

The current study has several limitations. A major shortcoming is that the functions and mechanisms of MFAP2 were explored *in vitro*, they were needed to be further confirmed by *in vivo* and *in vitro* experiments. Secondly, in addition to TCGA and CGGA cohorts, another retrospective single-center cohort is necessary to validate the above findings. In this study, we have made a preliminary discovery that provides a basis for our next experiments to explore functional mechanisms.

## Conclusion

In conclusion, the current study verified the role of MFAP2 in malignant progression and immune microenvironment of glioma. MFAP2 overexpression is relevant to poor prognosis and is considered an independent factor in patients with glioma. Our findings reveal that MFAP2 may be a valuable prognostic marker for gliomas.

## Data Availability

The original contributions presented in the study are included in the article/Supplementary Material, and further inquiries can be directed to the corresponding authors.
