# Supplementary material for: Microfibrillar-associated protein 2 is a prognostic marker that correlates with the immune microenvironment in glioma
Source: Front Genet. 2022 Sep 20;13:989521. doi: 10.3389/fgene.2022.989521 (PMC9531167; doi:10.3389/fgene.2022.989521)
Supplement: Supplementary file 1 [file Table1.docx]

Supplementary Table S2 Correlation analysis between MFAP2 and related genes and markers of immune cells in Tumor Immune Estimation Resource (TIMER2.0).

| **Description** | **Gene markers** | **Glioma** | | | | | | | |
| --- | --- | --- | --- | --- | --- | --- | --- | --- | --- |
|  |  | **LGG** | | | | **GBM** | | | |
|  |  | **None** | | **Purity** | | **None** | | **Purity** | |
|  |  | **Cor** | **p** | **Cor** | **p** | **Cor** | **p** | **Cor** | **p** |
| CD8+T cell | CD8A | 0.244 | *** | 0.266 | *** | 0.275 | ** | 0.237 | * |
|  | CD8B | 0.124 | * | 0.137 | * | 0.063 | 0.440 | 0.010 | 0.910 |
| General T cell | CD2 | 0.403 | *** | 0.407 | *** | 0.117 | 0.150 | 0.070 | 0.417 |
|  | CD3D | 0.379 | *** | 0.395 | *** | 0.053 | 0.518 | -0.011 | 0.902 |
|  | CD3E | 0.384 | *** | 0.398 | *** | 0.163 | 0.044 | 0.130 | 0.130 |
| B cell | CD19 | 0.330 | *** | 0.329 | *** | 0.088 | 0.282 | 0.096 | 0.265 |
|  | CD79A | 0.223 | *** | 0.239 | *** | 0.280 | ** | 0.269 | * |
| Monocyte | CD86 | 0.231 | *** | 0.257 | *** | -0.011 | 0.891 | -0.017 | 0.842 |
|  | CSF1R | 0.130 | * | 0.150 | ** | 0.064 | 0.429 | 0.073 | 0.394 |
| TAM | CCL2 | 0.293 | *** | 0.300 | *** | 0.046 | 0.570 | 0.011 | 0.899 |
|  | CD68 | 0.253 | *** | 0.268 | *** | 0.187 | 0.021 | 0.219 | 0.010 |
|  | IL10 | 0.252 | *** | 0.260 | *** | 0.102 | 0.208 | 0.116 | 0.177 |
| M1 Macrophag | IRF5 | 0.218 | *** | 0.257 | *** | 0.041 | 0.616 | -0.021 | 0.808 |
|  | NOS2 | -0.011 | 0.807 | 0.008 | 0.857 | 0.035 | 0.664 | 0.076 | 0.379 |
|  | PTGS2 | 0.141 | * | 0.138 | * | 0.166 | 0.040 | 0.167 | 0.051 |
| M2 Macrophage | CD163 | 0.302 | *** | 0.298 | *** | 0.204 | 0.011 | 0.218 | 0.011 |
|  | MS4A4A | 0.239 | *** | 0.249 | *** | 0.194 | 0.016 | 0.253 | * |
|  | VSIG4 | 0.150 | ** | 0.158 | ** | 0.128 | 0.114 | 0.165 | 0.054 |
| Neutrophils | CCR7 | 0.261 | *** | 0.275 | *** | 0.271 | ** | 0.253 | * |
|  | CEACAM8 | 0.037 | 0.398 | 0.029 | 0.529 | 0.118 | 0.147 | 0.086 | 0.319 |
|  | ITGAM | 0.211 | *** | 0.240 | *** | 0.083 | 0.306 | 0.084 | 0.327 |
| Natural killer cell | KIR2DL1 | 0.127 | * | 0.122 | * | 0.020 | 0.810 | 0.013 | 0.880 |
|  | KIR2DL3 | 0.208 | *** | 0.215 | *** | 0.098 | 0.227 | 0.082 | 0.343 |
|  | KIR2DL4 | 0.208 | *** | 0.208 | *** | -0.017 | 0.839 | -0.047 | 0.585 |
|  | KIR2DS4 | 0.127 | * | 0.144 | * | 0.082 | 0.314 | 0.095 | 0.271 |
|  | KIR3DL1 | 0.097 | 0.028 | 0.102 | 0.025 | 0.060 | 0.462 | 0.088 | 0.306 |
|  | KIR3DL2 | 0.115 | * | 0.125 | * | 0.013 | 0.877 | 0.030 | 0.726 |
|  | KIR3DL3 | 0.034 | 0.445 | 0.043 | 0.352 | 0.034 | 0.681 | -0.025 | 0.775 |
| Dendritic cell | CD1C | 0.247 | *** | 0.247 | *** | 0.150 | 0.065 | 0.136 | 0.113 |
|  | HLA-DPA1 | 0.401 | *** | 0.412 | *** | 0.125 | 0.125 | 0.146 | 0.090 |
|  | HLA-DPB1 | 0.423 | *** | 0.435 | *** | 0.153 | 0.059 | 0.187 | 0.029 |
|  | HLA-DQB1 | 0.328 | *** | 0.335 | *** | 0.061 | 0.453 | 0.135 | 0.117 |
|  | HLA-DRA | 0.414 | *** | 0.425 | *** | 0.098 | 0.228 | 0.117 | 0.175 |
|  | ITGAX | 0.208 | *** | 0.238 | *** | 0.050 | 0.536 | -0.013 | 0.883 |
|  | NRP1 | 0.329 | *** | 0.314 | *** | 0.415 | *** | 0.447 | *** |
| Th1 | IFNG | 0.154 | ** | 0.174 | ** | 0.072 | 0.377 | 0.027 | 0.750 |
|  | STAT1 | 0.356 | *** | 0.354 | *** | -0.119 | 0.144 | -0.131 | 0.126 |
|  | STAT4 | 0.050 | 0.257 | 0.063 | 0.166 | 0.090 | 0.270 | 0.055 | 0.522 |
|  | TBX21 | 0.408 | *** | 0.395 | *** | 0.411 | *** | 0.381 | *** |
|  | TNF | 0.054 | 0.219 | 0.044 | 0.339 | 0.143 | 0.078 | 0.094 | 0.273 |
| Th2 | GATA3 | 0.410 | *** | 0.431 | *** | 0.172 | 0.034 | 0.103 | 0.232 |
|  | STAT5A | 0.323 | *** | 0.350 | *** | 0.087 | 0.286 | 0.077 | 0.368 |
|  | STAT6 | 0.199 | *** | 0.262 | *** | 0.338 | *** | 0.342 | *** |
| Th17 | IL17A | -0.009 | 0.845 | -0.013 | 0.769 | 0.087 | 0.284 | 0.092 | 0.285 |
|  | STAT3 | 0.350 | *** | 0.328 | *** | 0.044 | 0.592 | 0.055 | 0.521 |
| Treg | CCR8 | 0.154 | ** | 0.162 | ** | 0.187 | 0.020 | 0.162 | 0.059 |
|  | FOXP3 | -0.058 | 0.188 | -0.048 | 0.300 | 0.047 | 0.566 | -0.013 | 0.878 |
|  | STAT5B | -0.029 | 0.512 | -0.038 | 0.413 | 0.004 | 0.956 | 0.014 | 0.873 |
|  | TGFB1 | 0.300 | *** | 0.315 | *** | 0.369 | *** | 0.384 | *** |
| T cell exhaustion | CTLA4 | 0.246 | *** | 0.251 | *** | 0.156 | 0.054 | 0.117 | 0.174 |
|  | GZMB | 0.349 | *** | 0.348 | *** | 0.068 | 0.403 | 0.052 | 0.543 |
|  | HAVCR2 | 0.266 | *** | 0.295 | *** | 0.001 | 0.991 | -0.024 | 0.779 |
|  | LAG3 | 0.284 | *** | 0.292 | *** | 0.246 | * | 0.213 | 0.012 |
|  | PDCD1 | 0.339 | *** | 0.339 | *** | 0.281 | ** | 0.258 | * |
|  | PDCD1LG2 | 0.345 | *** | 0.371 | *** | -0.086 | 0.293 | -0.068 | 0.428 |

Note. LGG, low-grade glioma. GBM, glioblastoma. TAM, tumor-associated macrophage. Th, T helper cell. Treg, regulatory T cell. Cor, R value of Spearman’s correlation. None, correlation without adjustment; Purity; correlation adjusted by purity. *p < 0.01; **p < 0.001; ***p < 0.0001.
